# Editorial: Immunotherapeutic advances in brain tumours

**DOI:** 10.3389/fimmu.2026.1949875

**Published:** 2026-08-12

**Authors:** Terry Lichtor, Insug O-Sullivan, Bingtao Tang

**Affiliations:** 1 Department of Neurosurgery, Rush University Medical Canter, Chicago, IL, United States; 2 University of Illinois Chicago, Chicago, IL, United States; 3 San Biomedical Tech Inc., Yongkang, China

**Keywords:** brain oncology, brain tumour, brain tumour immunology, gene therapy, immunotherapy

The convergence of immunological science and oncological practice has yielded transformative approaches for combatting cancers, including malignancies arising in anatomically challenging sites like the brain. Although the central nervous system has long been regarded as a sanctuary shielded from immune surveillance, emerging evidence substantiates the feasibility of immunotherapeutic interventions for both intrinsic cerebral neoplasms and metastatic lesions. Modalities encompassing dendritic cell-based vaccines loaded with tumour-associated antigens or messenger RNA, alongside checkpoint blockade agents that disarm inhibitory signalling pathways, represent compelling avenues for amplifying anti-tumour immunity.

The molecular distinctions between healthy and neoplastic cells within an individual provide the conceptual foundation for immunotherapy-based clinical interventions. Even though the brain has historically been classified as an immune-protected organ, a growing body of experimental work illustrates that immunotherapeutic modalities may hold therapeutic value for managing both intrinsic and metastatic brain cancers. Vaccination using dendritic cells pulsed with tumour lysates or engineered to express tumour-derived RNA can elicit robust CD8+ cytotoxic T lymphocyte (CTL) responses capable of recognizing and eliminating autologous malignant cells. Complementary investigations have explored immune checkpoint blockade, wherein antibodies neutralize co-inhibitory receptors that normally restrain T-cell activation, thereby reinvigorating anti-tumour immunity across diverse malignancies, including those of the central nervous system. While preclinical animal studies with dendritic cell vaccines and checkpoint inhibitors have yielded encouraging outcomes, human trials have frequently revealed transient clinical benefits confined to a subset of recipients. In highly aggressive entities such as gliomas, tumour progression is facilitated by a suppressive microenvironment characterized by enrichment of regulatory T cells (Treg) and myeloid-derived suppressor cells (MDSC). Furthermore, cytokine-encoded gene vaccines utilizing IL-15 or IL-2 have demonstrated the capacity to provoke vigorous anti-tumour immune reactions and extend survival in rodent models of brain cancer.

The overarching objective of oncological treatment is the complete eradication of all residual malignant cells from the host organism. Achieving this ambitious target through a single therapeutic modality alone appears improbable. Nevertheless, immunotherapy—integrated alongside established modalities such as surgical resection, radiotherapy, and cytotoxic chemotherapy—is poised to emerge as a valuable adjunctive approach in the neuro-oncology field. Even though immunotherapeutic interventions have shown considerable efficacy against multiple tumour types, their application to brain malignancies has produced variable outcomes with contradictory findings across studies. There is an undeniable and pressing need for deeper investigation in this domain. For this Research Topic, we invite contributions that examine the therapeutic promise of diverse immunotherapeutic approaches for brain tumour management and related facets of cancer immunology.

This Research Topic is dedicated to advancing the frontier of immune-based therapeutics for brain tumours, with the objectives of elucidating underlying mechanisms, optimizing therapeutic potency, and expanding the clinical applicability of these treatments. In particular, we seek studies investigating how innovative immunotherapeutic platforms might circumvent the immunosuppressive tumour microenvironment of gliomas and other formidable central nervous system malignancies, ultimately generating durable and robust immune reactions against these challenging adversaries.

Gao et al., in a review entitled, “Emerging frontiers in glioma therapy: the evolving role of glioma vaccines”, emphasized that successful design of glioma vaccines should include biological, immunological and clinical aspects.

In this review, they provide an integrative overview of current glioma vaccine strategies, including peptide-based, dendritic cell-based, nucleic acid-based, and autologous tumour-derived platforms, with a particular focus on their biological rationale, clinical performance, and translational limitations. They highlight forward-looking design principles for next-generation glioma vaccines, including improved antigen selection, biomarker-guided patient stratification, microenvironment-aware therapeutic design, and more predictive translational models. Overall, they propose that future progress in glioma vaccine therapy will depend not only on improving vaccine platforms themselves, but also on integrating them into biologically rational and clinically informed therapeutic frameworks.

Zhai et al., in a review, “Chimeric antigen receptor macrophages therapy for glioblastoma: challenges and opportunities from preclinical evidence to clinical translation” proposed that macrophages bearing chimeric antigen receptor would be the promising future for glioma therapy.

Treatment failure in glioblastoma (GBM) is primarily attributed to the convergence of multiple barriers, including an immunosuppressive tumour microenvironment (TME), intratumoural heterogeneity, and the blood-brain barrier. Chimeric antigen receptor macrophages (CAR-M) therapy presents a promising new avenue for GBM treatment, leveraging its inherent tumour-homing capacity, TME reprogramming function, and potential to bridge innate and adaptive immunity. This review aims to provide a systematic and critical reference to guide the translation of CAR-M therapy from concept to clinical application, a path characterized by both opportunities and challenges.

Kiel et al., in a review, “Harnessing immunotherapy: cancer vaccines as novel therapeutic strategies for brain tumour” foresaw that brain cancer therapy would fuse universal vaccines with personalized immunotherapy.

Recent advancements in antigen identification and sequencing techniques have catalysed the development of cancer vaccines whose goal is to elicit robust humoral and cellular immune responses against cancer cells. Despite their potential, most cancer vaccines are still in the experimental phase, primarily due to challenges associated with tumour-induced immune suppression. This article explores the role of cancer vaccines in brain cancer, glioblastoma, by providing a granular analysis of clinical trial results and mechanisms of resistance alongside a comparative assessment. This review highlights the potential impact of cancer vaccine clinical trials on future cancer therapies, where effective anti-cancer strategies are within reach. It also provides an in-depth discussion of the brain tumour microenvironment and its influence on vaccine efficacy.

Huang et al., in a case report, “Intrathecal administration of PD-1 inhibitor combined with pemetrexed for leptomeningeal metastases from breast cancer: a case report”, presented a promising combination of immunotherapy with chemotherapy.

Leptomeningeal metastasis (LM) is a fatal complication of malignant tumours with limited treatment options. This case reports an LM patient from breast cancer treated with intrathecal pemetrexed (15 mg) combined with PD-1 inhibitors (40 mg). The patient showed good tolerance, with no severe adverse events observed, and achieved favourable therapeutic outcomes, including complete resolution of neurological symptoms, negative conversion of cerebrospinal fluid cytology, and significant reduction of imaging-detected lesions. This case provides a new approach to the treatment of LM, suggesting that intrathecal immunotherapy combined with intrathecal chemotherapy may be a safe and effective treatment option, offering valuable insights for future clinical applications.

Braitbard et al., in a research report, “Maternal immunization impairs lymphoma growth and CNS/ocular metastasis in the offspring”, demonstrated that the immunity can be transferred through the maternal milk.

Maternal immunization is an important tool directed against a variety of infectious maladies in the offspring. Ocular lymphoma is a lethal disease caused mainly by two clinically distinct forms of non-Hodgkin’s lymphoma: non-Hodgkin’s lymphoma of the central nervous system, or primary CNS lymphoma (PCNSL), and systemic lymphoma metastatic to the eye. Previously, they developed an experimental model whereby mouse lymphoma cell variants, derived from the S49 T-cell lymphoma, metastasized to the CNS and eyes following intraperitoneal inoculation at days 7–10 postnatal. Here, they extended the model to study whether maternal immunization can impede CNS/Ocular metastasis in the offspring exposed to the metastatic lymphoma cells. Immunity was conferred via milk suckling and was prolonged without further challenge for an extended period of at least 3 months. The abovementioned findings constitute a novel experimental model system whereby CNS/Ocular metastasis of malignant lymphoma in the offspring is impeded through maternal vaccination/immunization and thus, can be followed mechanistically as well as for novel therapeutic modalities.

Li et al., in a review, “Decoding the immune microenvironment: precision immunotherapy for medulloblastoma subtypes”, correlated the heterogeneity analysis with the improved precision therapy.

Medulloblastoma is a severe paediatric brain tumour with distinct molecular subtypes—WNT, SHH, Group 3, and Group 4-each having unique genetic drivers and immune microenvironments. This review highlights the immune characteristics of each subtype: SHH is rich in tumour-associated macrophages (TAMs), whose role in tumourigenesis is debated; Group 3 features cytotoxic T cells often neutralized by immune checkpoints like PD-L1, causing T cell exhaustion; and Group 4 is marked by natural killer (NK) cells and B cells. Understanding the immune microenvironment’s subtype-specific heterogeneity in medulloblastoma is crucial for advancing precision immunotherapy and improving patient outcomes.

Ali Khan Saddozai et al., in a research report, “Prognostic value of metal-based ferroptosis and cuproptosis genes and score in lower grade gliomas”, suggested the improved immunotherapy with iron and copper induced cell death.

Ferroptosis and Cuproptosis are newly defined forms of cell death. Despite distinct mechanisms, both involve metabolic processes in the TCA cycle and downstream pathways, crucial for anticancer immunity. They evaluated Iron (Fe) and Copper-induced cell death in lower-grade gliomas (LGG) using The Cancer Genome Atlas (TCGA) data by developing a metal-based ferroptosis and cuproptosis genes score (MBFCGs) risk model. The MBFCGs risk model is a promising prognostic tool for LGG, offering insights into underlying mechanisms and new directions for immunotherapy strategies. Assessment of MBFCGs for individual LGG patients may provide clues for developing new immunotherapy strategies.

Zhu et al., in a review, “Research progress on the role of dendritic cells in glioma during 1992-2024: a bibliometric analysis”, suggested dendritic cell-based glioma vaccines can be improved by resolving immunosuppression.

The extracted literature related to glioma and dendritic cells from 1992 to 2024 using the Web of Science Core Collection. Utilizing Cite Space, Vos viewer and Microsoft Excel, they analysed the volume of publications, the contributing countries/regions, institutions, authors, journals, references and keywords. The findings suggest that dendritic cells, immunotherapy and glioblastoma treatment will remain the focal points and emerging trends in dendritic cell-glioma research, providing valuable insights for future studies. Dendritic cell vaccines show promise in glioma trials but are hindered by the immunosuppressive tumour microenvironment.

Wang et al., in a research report, “scRNA-seq unveils the functional characteristic of glioma-associated macrophages and the regulatory effect of chlorogenic acid on the immune microenvironment-a study based on mouse models and clinical practice”, chlorogenic acid (CHA) was used in model mice in this study, and scRNA - seq analysis was conducted to elucidate the differentiation trajectories and functional characteristics of bone marrow-derived mono-macrophages (BMDMs) and microglia.

A PPI and molecular docking model were constructed using the target prediction database. A case of a patient treated with CHA was reviewed. This study may provide new insights into targeting the regulation of tumour microenvironment (TME) and offer theoretical and practical support for the clinical application of CHA. The results demonstrated the potential of CHA in improving the immune microenvironment and antitumour effects, which could have implications for future glioma treatment strategies. They conclude that the JAK-STAT pathway is the core molecular link between myeloid cells, microglia in the glioma microenvironment, and the action mechanism of CHA.

